# Pharmacotherapy of atopic eczema - an analysis of the discrepancies between recent expert guidelines, official drug licenses, and evidence for efficacy of recommended drugs

**DOI:** 10.1186/2045-7022-5-S1-P4

**Published:** 2015-03-11

**Authors:** Katarzyna Kordus, Radoslaw Spiewak

**Affiliations:** 1 Jagiellonian University Medical College, Department of Experimental Dermatology and Cosmeto, Krakow, Poland

## 

The Polish Physician's and Dentist's Profession Act and Polish Pharmaceutical Law oblige physicians to prescribing drugs strictly in line with the official indications listed in the Summaries of Product Characteristic (SPC). Prescriptions beyond SPC ('off-label') may be interpreted as ‘medical experiments’ with legal and financial liability resting solely with the doctor, the reimbursement is also denied. The aimof the study was to analyze discrepancies between up-to-date expert guidelines for treating atopic eczema (AE), indications listed in SPC, reimbursement policy, and scientific evidence for the efficacy of recommended drugs. Expert recommendations for the treatment of AE were confronted with SPC of recommended drugs and their reimbursement scheme. A systematic review of clinical trials was done, with their quality assessed using the GRADE tool. Among drugs recommended by experts for the treatment of AE, 484 medicinal products were licensed for use in Poland, including 89 with official indication for AE. Of these, clinical trials confirmed efficacy of active components of 36 topical or systemic calcineurin inhibitors and 19 topical glucocorticoid preparations. Thirty preparations for AE were reimbursed, including 19 recommended by experts and confirmed effective. Further 75 products with active substances recommended and effective were licensed for use in Poland, but not for AE. We conclude that in the pharmacotherapy of atopic eczema there are considerable discrepancies between expert recommendations, scientific evidence for the effectiveness of recommended drugs, and the acceptable uses of the drugs determined by the Summaries of Product Characteristics.

